# Skin and nasal colonization of coagulase-negative staphylococci are associated with atopic dermatitis among South African toddlers

**DOI:** 10.1371/journal.pone.0265326

**Published:** 2022-03-17

**Authors:** Gillian O. N. Ndhlovu, Felix S. Dube, Rasalika T. Moonsamy, Avumile Mankahla, Carol Hlela, Michael E. Levin, Nonhlanhla Lunjani, Adebayo O. Shittu, Shima M. Abdulgader

**Affiliations:** 1 Department of Molecular and Cell Biology, Faculty of Science, University of Cape Town, Cape Town, South Africa; 2 Institute of Infectious Disease & Molecular Medicine, University of Cape Town, Cape Town, South Africa; 3 Department of Medicine and Pharmacology, Division of Dermatology, Walter Sisulu University, Umtata, South Africa; 4 Department of Paediatric, Division of Paediatric Allergy, University of Cape Town, Cape Town, South Africa; 5 Department of Microbiology, Obafemi Awolowo University, Ile-Ife, Osun State, Nigeria; 6 Institute of Medical Microbiology, University Hospital Münster, Münster, Germany; 7 Department of Biomedical Sciences, Division of Molecular Biology and Human Genetics, Faculty of Medicine and Health Sciences, Stellenbosch University, Tygerberg, South Africa; King Faisal University, SAUDI ARABIA

## Abstract

**Background:**

Skin colonization with coagulase-negative staphylococci (CoNS) is generally beneficial, but recent investigations suggest its association with flares and atopic dermatitis (AD) severity. However, this relationship remains unclear.

**Objective:**

To assess patterns of staphylococcal colonization and biofilm formation in toddlers with and without AD from rural and urban South African settings.

**Methods:**

We conducted a cross-sectional study of AD-affected and non-atopic AmaXhosa toddlers from rural Umtata and urban Cape Town, South Africa. CoNS isolates were recovered from lesional, nonlesional skin samples and the anterior nares of participants. Identification of the staphylococci was achieved by MALDI-TOF mass spectrometry. The microtiter plate assay assessed *in-vitro* biofilm formation.

**Results:**

CoNS and *S*. *aureus* commonly co-colonized nonlesional skin among cases (urban: 24% vs. 3%, p = 0.037 and rural 21% vs. 6%, p<0.001), and anterior nares in urban cases (24% vs. 0%, p = 0.002) than the control group. *S*. *capitis* colonization on nonlesional skin and anterior nares was positively associated with more severe disease in rural (48.3±10.8 vs. 39.7±11.5, *P* = 0.045) and urban cases (74.9±10.3 vs. 38.4±13, *P =* 0.004), respectively. Biofilm formation was similar between cases and controls, independent of rural-urban living.

**Conclusion:**

CoNS colonization is associated with AD and disease severity and may be implicated in AD exacerbations. Studies are needed to understand their underlying pathological contribution in AD pathogenesis.

## Introduction

The skin is the first line of defense against the entry of pathogens. It is populated by a diverse community of bacteria, viruses, fungi, and mites [1]. The genus *Staphylococcus* represents the largest bacterial group on healthy skin and anterior nares [2, 3]. Moreover, the coagulase-negative staphylococci (CoNS) comprising *Staphylococcus epidermidis*, *Staphylococcus hominis*, and *Staphylococcus haemolyticus* are the most dominant species [4, 5]. CoNS are skin commensals that extensively interact with host epidermal and immune cells to maintain the skin homeostasis and protect against opportunistic infections [6]. They competitively prevent *Staphylococcus aureus* colonization on healthy skin and anterior nares by producing various bacteriocins and antimicrobial peptides (AMPs) [6]. CoNS may antagonize *S*. *aureus* colonization through glycerol fermentation [7, 8] and synergizing with host AMPs [6]. CoNS can also inhibit the expression of *S*. *aureus* virulence factors, thereby limiting its pathological colonization [6].

Atopic dermatitis (AD) is a chronic inflammatory skin disease characterized by red, intensely itchy, and dry, inflamed skin lesions, often with an altered skin microbial community compared to healthy individuals [9]. *S*. *aureus* is the primary bacterial pathogen associated with AD disease flares [10]. Studies have shown that the frequency and relative abundance of CoNS with anti-*S*. *aureus* activity is reduced in AD due to the over-proliferation of *S*. *aureus* [11]. Sometimes, the proliferation of CoNS during AD flares coincides with *S*. *aureus* overgrowth [3]. Here, CoNS are proposed to compensate for the increase in *S*. *aureus* colonization or synergize with *S*. *aureus* leading to augmented *S*. *aureus* growth and pathological potential [3, 12, 13]. However, there are conflicting findings on the shifts in CoNS colonization and prevalence during AD flares than healthy skin and their correlation with AD severity in children and adults [14–17]. Although CoNS have a much lower pathological potential than *S*. *aureus*, antigens of *S*. *haemolyticus* and *S*. *epidermidis* can induce dysfunctional immune responses that contribute to chronic skin damage in AD [18, 19]. Also, some *S*. *epidermidis* strains contribute to the pathogenesis of AD through the production of the cysteine protease EcpA, which promotes epidermal damage and inflammation [19]. Furthermore, a recent study provided evidence of an increased abundance of *S*. *epidermidis* and *S*. *hominis* in Netherton Syndrome and ichthyosis vulgaris [14], highlighting the potential pathological role of CoNS in skin diseases. In this regard, research on AD is rapidly shifting towards understanding how CoNS may contribute to its pathogenesis.

The disrupted skin barrier in AD provides a unique environment that enhances staphylococcal binding through the exposure of epidermal extracellular matrix components [20, 21]. This scenario consequently triggers the formation of biofilms, a key determinant for the chronicity of *S*. *aureus* and CoNS colonization on AD skin [21]. Also, the putative pro-inflammatory environment in AD promotes staphylococcal biofilm growth [22]. *S*. *aureus* and *S*. *epidermidis* biofilms are generally present on AD skin and are associated with disease severity [22–24]. *In vitro* studies have shown that *S*. *epidermidis* may antagonize [25] or cooperate [26, 27] with *S*. *aureus* in biofilm formation. These studies on the interactions of CoNS in mixed biofilms with *S*. *aureus* have primarily included healthy individuals [12, 25, 28] and are limited in patients with AD [26]. Moreover, no study has compared these interactions between AD patients and healthy individuals to assess whether they differ based on AD disease status.

We explored the skin and nasal staphylococcal colonization patterns in AD cases and healthy controls across urban and rural environments. We also evaluated biofilm formation of staphylococcal species, including *S*. *aureus* and CoNS interactions in mixed-species biofilms.

## Materials and methods

### Study population

We recruited 220 AmaXhosa (same ethnolinguistic background) toddlers aged 9–38 months (mean, 22.5 months; standard deviation, 7.3 months) with and without AD from Umtata, South Africa, and Cape Town, South Africa. The duration of the recruitment was between February 2015 and May 2016. Toddlers with an AD diagnosis based on the United Kingdom Working Party diagnosis of atopic eczema [29] were recruited through the Red Cross Children’s War Memorial Hospital (Cape Town, South Africa) and Nelson Mandela Academic Hospital (Umtata, South Africa). AD severity was measured, at the time of specimen collection, using the objective scoring of atopic dermatitis (SCORAD) index [30]. Similarly, aged toddlers without a clinical diagnosis of AD were recruited from the community early development centers in Cape Town and Umtata. Swabs were collected from the anterior nares and nonlesional skin (area with most normal-appearing skin–usually the back) in both cases and controls as previously described [31]. We also collected lesional skin swabs (i.e., most active area of eczematous skin with acute and or chronic changes) from only cases. Collected swabs were placed in STGG and stored at -80°C for subsequent batch processing.

### Bacterial isolation and species identification

To recover the CoNS isolates, 20μL of each specimen was inoculated on mannitol salt agar (National Health Laboratory Services [NHLS], South Africa), streaked for single colony growth, and incubated at 37°C in ambient air for 48 hours. All morphologically distinct colonies were selected for further analysis. Species identification was performed by matrix-assisted laser desorption ionization time-of-flight (MALDI-TOF) mass spectrometry. All identified CoNS isolates were stored at -20°C in skim milk-tryptone-glucose-glycerine (STGG) medium (NHLS, South Africa) for further batch processing. *S*. *aureus* isolates included in the analyses within this study were recovered previously [31].

### Assessment of in vitro mono-species and dual-species biofilm formation

Biofilm formation was assessed *in vitro* using the crystal violet microtiter assay (32). Staphylococcal isolates in STGG were streaked on 2% blood agar and incubated overnight at 37°C in ambient air. A single colony was inoculated into 3mL 3% tryptic soy broth supplemented with 0.1% glucose (gTSB; Merck, South Africa) followed by incubation with shaking for 24 hours. Thereafter, the broth culture was diluted at 1:100 for mono-species biofilms and 100μL:100μL for dual staphylococcal biofilms in a flat-bottomed 96-well microtiter plate (Lasec, South Africa) previously described [26]. Then, the plates were statically incubated in ambient air at 37°C for 24 hours. *S*. *aureus* ATCC 29213 and uninoculated gTSB were included as positive and negative controls, respectively. Planktonic bacterial suspensions were discarded, followed by rinsing the microtiter plates three times in sterile reverse osmosis (RO)-filtered water and dried at 60°C for 1 hour. The formed biofilms were stained with 200μL 0.1% crystal violet and incubated at room temperature for 10 minutes and followed by discarding excess crystal violet. Thereafter, the stained biofilms were solubilized with 200μL 30% acetic acid and incubated at room temperature for 30 minutes. The absorbance was measured at 492nm using the Mindray MR 96A ELISA Microplate Reader (Vacutec, South Africa). Biofilm biomass was analyzed using OD readings as previously described (OD>OD negative control [OD_c_], non-producer; OD_c_<OD<2·OD_c_, weak producer; 2·OD_c_<OD<4·OD_c_, moderate producer; and OD>4·OD_c_, strong producer) [32].

### Statistical analysis

We used R studio version 4.0.4 to conduct statistical analyses. Species with less than ten isolates were arbitrarily considered rare in this cohort and classified as “rare CoNS.” The Fisher’s exact test was performed for comparisons between categorical variables. Differences in continuous dependent variables by a categorical variable were assessed using either the Wilcoxon rank-sum test or the Kruskal-Wallis test. We used the Bonferroni method to adjust for multiple comparisons. All analyses were two-tailed. The log2 fold change method was used to compare the fold change of the biofilm biomass from dual CoNS-*S*. *aureus* biofilms compared to mono-species *S*. *aureus* biofilms. The effect on biofilm biomass was arbitrarily classified based on log2 fold change as follows: “no effect” (> -0.1 to <0.1); “weak, positive” (0.1 to <0.4); “moderate, positive” (0.4 to <0.9); “strong, positive” (>0.9); “weak, negative” (-0.1 to < -0.4); “moderate, negative” (-0.4 to < -0.9). Correlation between continuous variables was assessed using Pearson’s correlation. A *p*-value of <0.05 was considered statistically significant.

## Statement of ethics

The parent study was approved by the Human Research and Ethics Committee of the Faculty of Health Science, University of Cape Town (HREC/REF: 451/2014) and the Western Cape Provincial Child Health Research Committee. Additional ethical approval specifically for the study was obtained from the Human Research and Ethics Committee of the Faculty of Health Science, University of Cape Town (HREC/REC: 668/2020). No additional data was collected other than that approved in the parent study. Written informed consent and assent were provided by guardians and participants, respectively. All data obtained and generated during the study were kept confidential. This research was conducted in accordance with the Declaration of Helsinki.

## Results

### Risk factors and staphylococcal colonization patterns

**Fig 1:**
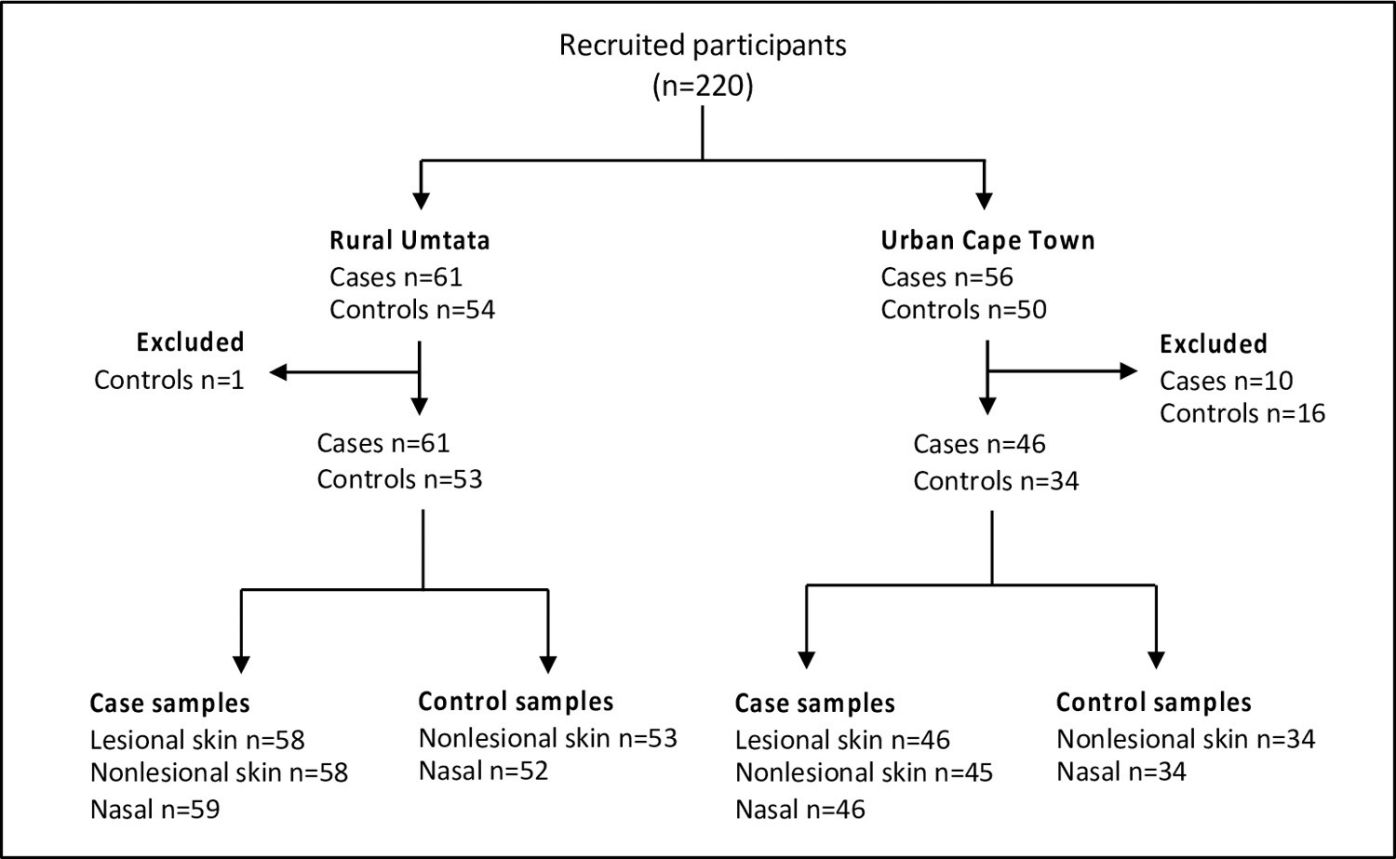
Flow chart of study participant specimens included in the analyses. Twenty-seven participants were excluded from analysis due to the unavailability of samples.

Table 1 describes the characteristics of the participants. We recovered 381 staphylococcal isolates from lesional skin (n = 112), nonlesional skin (cases: n = 117; controls: n = 60) and anterior nares (cases: n = 67; controls: n = 25) from cases and controls (Fig 1). These isolates represented 16 different staphylococcal species including *S*. *aureus* (n = 129), *S*. *epidermidis* (n = 104), *S*. *hominis* (n = 53), *S*. *haemolyticus* (n = 38), *S*. *capitis* (n = 28), *S*. *saprophyticus* (n = 10), *S*. *warneri* (n = 5), *S*. *cohnii* (n = 4), *S*. *equorum* (n = 2), *S*. *pasteuri* (n = 2), *S*. *caprae* (n = 1), *S*. *lentus* (n = 1), *S*. *lugdunensis* (n = 1), *S*. *nepalensis* (n = 1), *S*. *sciuri* (n = 1), and *S*. *succinus* (n = 1). Antibiotic exposure (aOR [95% CI, 5.25 [1.68–16.38]) and having AD (4.67 [1.58–13.77]) are risk factors for CoNS colonization among urban toddlers (S1 Table).

**Table 1:** Participant characteristics.

	AD cases	non-AD controls
No. of participants	107	87
Male, %	54 (58/104) ^a^	59 (51/87)
Rural, %	57 (61/107)	61 (53/87)
Age, mean (range)	22.39 (9–38)	22.61 (12–36)
Objective SCORAD, mean (range)	41.51 (21.4–82.2)	—
No. of staphylococcal isolates %	78 (296/381)	22 (85/381)

^a^ Data of participant sex is not available for all analyzed participants.

**Fig 2:**
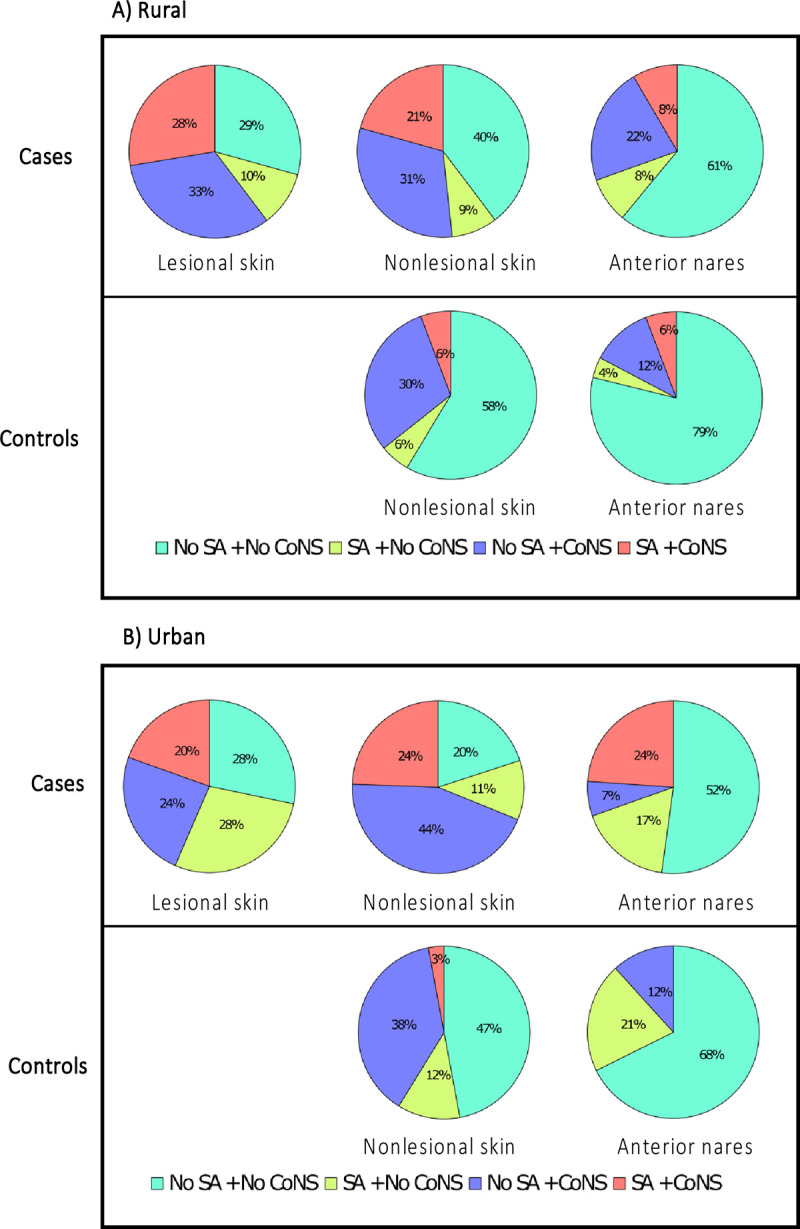
The distribution of *S*. *aureus* and CoNS colonization on the lesional, nonlesional skin, and anterior nares of (A) rural and (B) urban cases and controls. CoNS colonization is defined as the carriage of at least one CoNS species. SA, *S*. *aureus*; CoNS, coagulase-negative staphylococci.

The distribution of staphylococcal colonization in cases and controls by geographic location is shown in Fig 2. Colonization with only *S*. *aureus*, CoNS, or both was common on lesional skin in the rural and urban cohorts. In the urban cohort, 47% (16/34) of controls had no staphylococcal colonization on nonlesional skin compared to the nonlesional skin of cases (20% [9/45], *P* = 0.015). Co-colonization with CoNS species and *S*. *aureus* on nonlesional skin was more prevalent in cases from both rural (21% [21/58] vs. 6% [3/53], *P* <0.001) and urban (24% [9/45] vs. 3% [1/34], *P* = 0.037) settings than controls. Similarly, the colonization rates for only *S*. *aureus* or CoNS were higher on the nonlesional skin of cases than controls, independent of geographic location. Urban controls had no co-colonization with CoNS species and *S*. *aureus* in their anterior nares (Fig 2).

*S*. *haemolyticus* was only isolated from the nonlesional skin of rural cases versus the control group (10% [6/58) vs. 0% [0/58], *P* = 0.029). Moreover, *S*. *haemolyticus* prevalence was similar among lesional cases in the rural (10%) and urban (11%) cohorts (Tables 2 and 3). Also, *S*. *epidermidis* was significantly more frequent in the anterior nares of rural cases than controls (27% [16/59] vs. 10% [5/52], *P* = 0.029) (Table 2). However, the rare staphylococcal group was commonly found in the nonlesional skin of rural controls (11% [6/53] vs. 0% [0/58], *P* = 0.009) (Table 2). Overall, colonization with most CoNS species on AD lesional skin did not significantly differ from nonlesional skin and anterior nares in both geographic locations. However, a few exceptions were observed. *S*. *capitis* was more frequently reported in nonlesional skin (20% [9/45] vs. 3% [1/34], *P* = 0.038) and anterior nares (4.3% [2/46] vs. 0% [0/34]) of cases compared to controls in the urban cohort (Table 3). Comparing CoNS species between sampling sites revealed a higher colonization prevalence of *S*. *hominis* in the nonlesional skin than lesional skin (*P* = 0.016) and anterior nares (*P* = 0.0004) in urban cases. This trend was also observed with *S*. *capitis* nonlesional skin colonization compared to anterior nares (*P* = 0.027) but not AD lesional skin (*P* = 0.069) among urban cases.

**Table 2:** CoNS species identified on the skin and anterior nares of Umthatha (rural) cases and controls.

Species	Lesional skin	Nonlesional skin			Anterior nares		
	Case, *n* (%) N = 58	Case, *n* (%) N = 58	Control, *n* (%) N = 53	p-value	Case, *n* (%) N = 59	Control, *n* (%) N = 52	p-value
*S*. *epidermidis*	24 (41.4)	17 (29.3)	8 (15.1)	0.116	16 (27.1)	5 (9.6)	**0.029**
*S*. *capitis*	3 (5.2)	7 (12.1)	1 (1.9)	0.066	2 (3.4)	0 (0)	0.498
*S*. *haemolyticus*	6 (10.3)	6 (10.3)	0 (0)	**0.029**	2 (3.5)	0 (0)	0.498
*S*. *hominis*	6 (10.3)	8 (13.8)	10 (18.9)	0.448	1 (1.7)	2 (3.8)	0.597
*S*. *saprophyticus*	2 (3.4)	0 (0)	1 (1.9)	0.465	0 (0)	1 (1.9)	0.465
Rare CoNS^a^	4 (6.9)	0 (0)	6 (11.3)	**0.009**	2 (3.4)	1 (1.9)	1.000

^a^Rare CoNS include *S*. *caprae*, *S*. *cohnii*, *S*. *equorum*, *S*. *lentus*, *S*. *lugdunensis*, *S*. *nepalensis*, *S*. *pasteuri*, *S*. *sciuri*, *S*. *succinus* and *S*. *warneri*. Underlined bold text indicates statistical significance.

**Table 3:** CoNS species identified on the skin and anterior nares of Cape Town (urban) cases and controls.

Species	Lesional skin	Nonlesional skin			Anterior nares		
	Case, *n* (%) N = 46	Case, *n* (%) N = 45	Control, *n* (%) N = 34	p-value	Case, *n* (%) N = 46	Control, *n* (%) N = 34	p-value
*S*. *epidermidis*	10 (21.7)	10 (22.2)	3 (8.8)	0.140	8 (17.4)	3 (8.8)	0.338
*S*. *capitis*	3 (6.5)	9 (20)	1 (2.9)	**0.038**	2 (4.3)	0 (0)	0.505
*S*. *haemolyticus*	5 (10.9)	9 (20)	8 (23.5)	0.784	2 (4.3)	0 (0)	0.505
*S*. *hominis*	4 (8.7)	13 (28.9)	7 (20.6)	0.602	1 (2.2)	1 (2.9)	1.000
*S*. *saprophyticus*	0 (0)	4 (8.9)	1 (2.9)	0.388	1 (2.2)	0 (0)	1.000
Rare CoNS^a^	1 (2.2)	1 (2.2)	3 (8.8)	0.307	1 (2.2)	0 (0)	1.000

^a^Rare CoNS include *S*. *caprae*, *S*. *cohnii*, *S*. *equorum*, *S*. *lentus*, *S*. *lugdunensis*, *S*. *nepalensis*, *S*. *pasteuri*, *S*. *sciuri*, *S*. *succinus* and *S*. *warneri*. Underlined bold text indicates statistical significance.

### Colonization with CoNS is associated with AD severity

**Fig 3:**
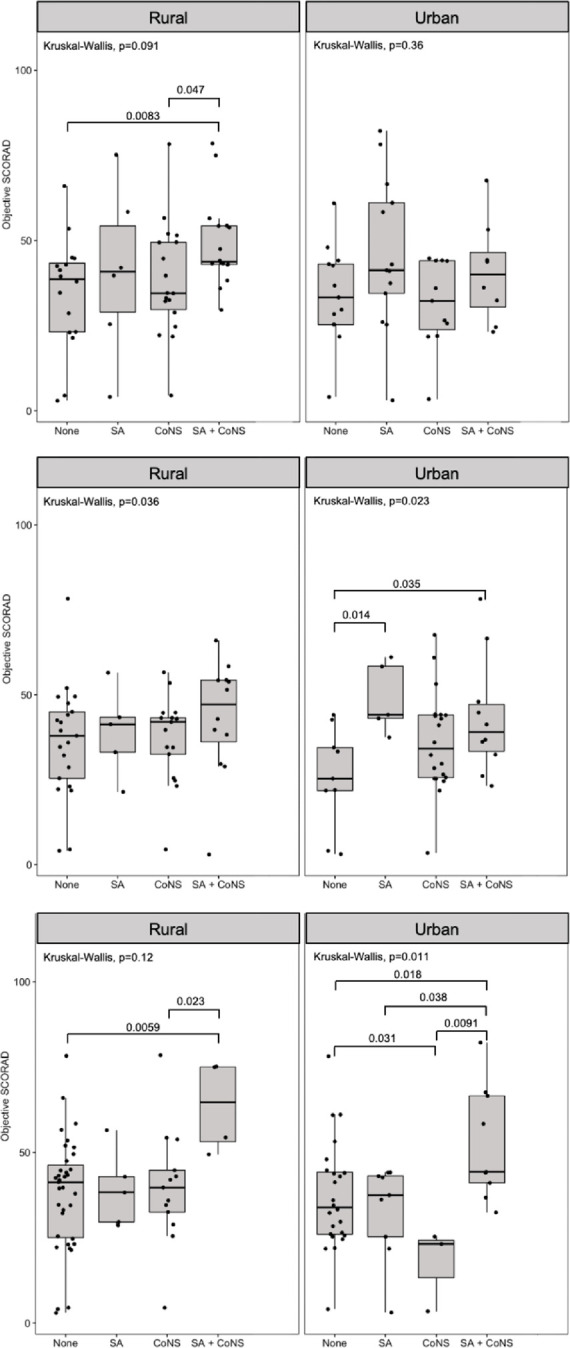
Relationship between objective SCORAD and staphylococcal colonization on lesional skin (upper panels), nonlesional skin (middle panels), and anterior nares (lower panels). None, no staphylococcal colonization; SA, *Staphylococcus aureus*; CoNS, coagulase-negative *Staphylococcus*; SA + CoNS, simultaneous colonization with *S*. *aureus* and CoNS.

AD severity, as determined by objective SCORAD scores, was highest amongst rural cases co-colonized by *S*. *aureus* and CoNS on lesional skin (median [IQR], 54.5 [49.5–71.5]). This when was compared to those without staphylococcal colonization (40.5 [18–54.5], *P* = 0.0083), and those by CoNS only (35 [23–65], *P* = 0.047) (Fig 3, upper panel). A similar pattern of higher severity scores was observed among rural cases co-colonized with *S*. *aureus* and CoNS in their nonlesional skin compared to those without colonization. However, it did not reach statistical significance (Fig 3, middle panel). In urban cases, lower severity scores were associated with the absence of staphylococcal colonization on nonlesional skin (28 [9–41]) compared to cases colonized with *S*. *aureus* only (56 [51–75], *P* = 0.014), and to those co-colonized with *S*. *aureus* and CoNS (39.5 [25–63], *P* = 0.035) (Fig 3, middle panel). Higher AD severity scores were associated with co-colonization with *S*. *aureus* and CoNS in the anterior nares of cases from urban (58 [43–79] vs. 31 [13.5–57.5], *P* = 0.018) and rural (76.5 [68–81.5] vs. 44 [23–62], *P* = 0.0059) settings (Fig 3, lower panel). This was in comparison with no staphylococcal colonization in the anterior nares of participants. Moreover, urban cases showed lower AD severity scores when only colonized with CoNS (31 [13.5–57.5]) in their anterior nares compared to no colonization at all (9 [6–22], *P* = 0.031) (Fig 3, lower panel). When considering individual CoNS species, a significant positive association between higher objective SCORAD scores and *S*. *capitis* colonization was observed for the nonlesional skin of rural cases (48.3±10.8 vs. 39.7±11.5, *P* = 0.045, Table 4). This was also noted with the anterior nares of urban cases compared with those not colonized (74.9±10.3 vs. 38.4±13, *P =* 0.004, Table 5).

**Table 4:** Mean values of objective SCORAD based on the presence or absence of CoNS in rural cases.

Species	Lesional skin			Nonlesional skin			Anterior nares		
	Sample + *μ* ± SD	Sample– *μ* ± SD	p-value	Sample + *μ* ± SD	Sample– *μ* ± SD	p-value	Sample + *μ* ± SD	Sample– *μ* ± SD	p-value
*S*. *epidermidis*	45.2 ± 13.7	41.3 ± 14.1	0.209	39.9 ± 11.6	41.2 ± 11.9	0.931	47.1 ± 16.5	40.9 ± 12.7	0.283
*S*. *capitis*	48.8 ± 22.8	42.6 ± 13.5	0.967	48.3 ± 10.8	39.7 ± 11.5	**0.045**	64.4 ± 15	41.8 ± 13.8	0.061
*S*. *haemolyticus*	52.4 ± 16	41.8 ± 13.4	0.060	44 ± 10.6	40.4 ± 11.6	0.497	52 ± 32.7	42.3 ± 13.4	0.781
*S*. *hominis*	37.9 ± 9.2	43.6 ± 14.4	0.367	37.5 ± 6.4	41.3 ± 12.3	0.409	NA	42.5 ± 14.1	–
*S*. *saprophyticus*	40.8 ± 3.5	42.7 ± 14.1	0.918	NA	40.8 ± 13.7	–	NA	42.6 ± 13.9	–
Rare CoNS^a^	41.4 ± 16	43.1 ± 14	0.967	NA	40.8	–	NA	42.4 ± 14	–

NA represents observations that are too few for mean and standard deviation calculation. Sample +, CoNS present; Sample -, CoNS absent; *μ*, mean; SD, standard deviation. Underlined bold text indicates statistical significance. *P-*values were determined using the Wilcoxon rank-sum test.

^a^Rare CoNS include *S*. *caprae*, *S*. *cohnii*, *S*. *equorum*, *S*. *lentus*, *S*. *lugdunensis*, *S*. *nepalensis*, *S*. *pasteuri*, *S*. *sciuri*, *S*. *succinus* and *S*. *warneri*.

**Table 5:** Mean values of objective SCORAD based on the presence or absence of CoNS in urban cases.

Species	Lesional skin			Nonlesional skin			Anterior nares		
	Sample + *μ* ± SD	Sample– *μ* ± SD	p-value	Sample + *μ* ± SD	Sample– *μ* ± SD	p-value	Sample + *μ* ± SD	Sample– *μ* ± SD	p-value
*S*. *epidermidis*	37.5 ± 9	40.8 ± 16.2	0.951	37.7 ± 17.7	39.4 ± 12.6	0.318	40.1 ± 12.2	40 ± 15.7	0.678
*S*. *capitis*	46.1 ± 20.6	38.6 ± 13.1	0.366	40.7 ± 20.2	39.9 ± 13.6	0.818	74.9 ± 10.3	38.4 ± 13	**0.004**
*S*. *haemolyticus*	32.6 ± 9.2	39.7 ± 13.8	0.327	41.2 ± 17.9	39.8 ± 14.3	0.94	49.5 ± 24.2	39.6 ± 14.6	0.515
*S*. *hominis*	30.5 ± 10.6	39.9 ± 13.6	0.195	37.1 ± 11.6	41.3 ± 16	0.573	NA	40 ± 15	–
*S*. *saprophyticus*	NA	40 ± 14.9	–	34.8 ± 13.1	40.6 ± 15.1	0.554	NA	40.1 ± 15	–
Rare CoNS^a^	NA	39.3 ± 13.7	–	NA	40 ± 15	–	NA	40.3 ± 15	–

NA represents observations that are too few for mean and standard deviation calculation. Sample +, CoNS present; Sample -, CoNS absent; *μ*, mean; SD, standard deviation. Underlined bold text indicates statistical significance. *P-values* were determined using the Wilcoxon rank-sum test.

^a^Rare CoNS include *S*. *caprae*, *S*. *cohnii*, *S*. *equorum*, *S*. *lentus*, *S*. *lugdunensis*, *S*. *nepalensis*, *S*. *pasteuri*, *S*. *sciuri*, *S*. *succinus* and *S*. *warneri*.

### Biofilm propensity of staphylococcal species

**Fig 4:**
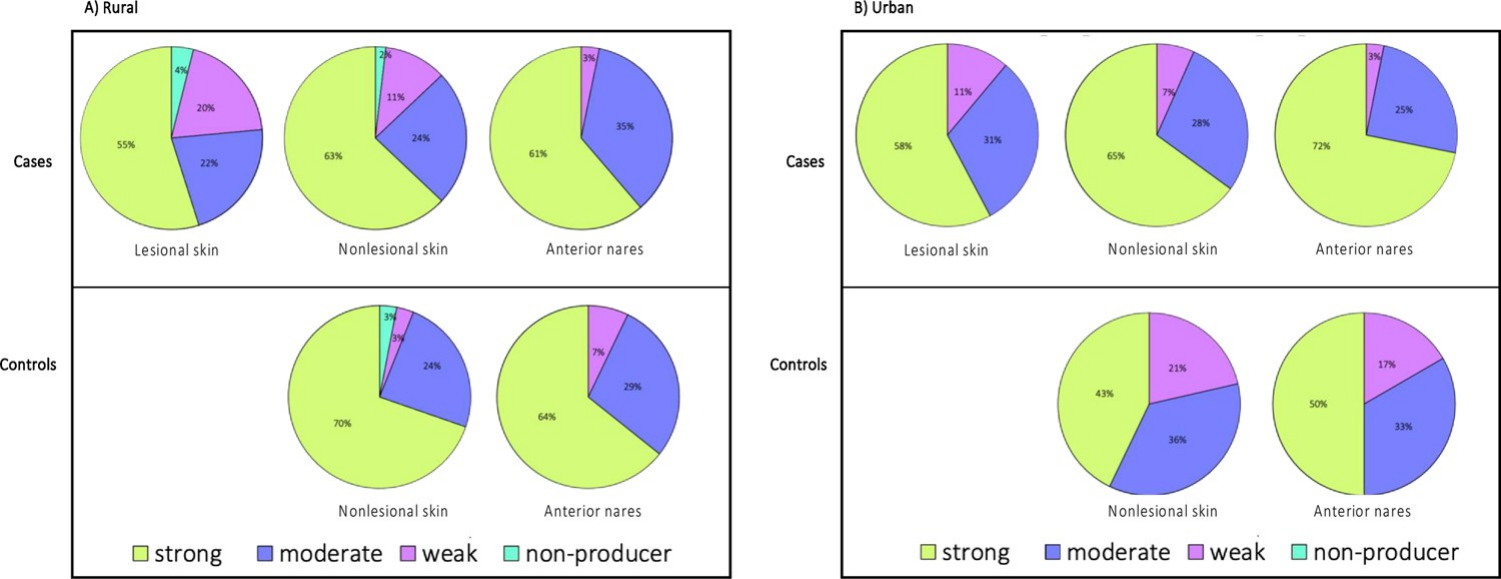
Prevalence of staphylococcal biofilm propensity in mono-species biofilms in (A) rural and (B) urban cases and controls.

**Fig 5:**
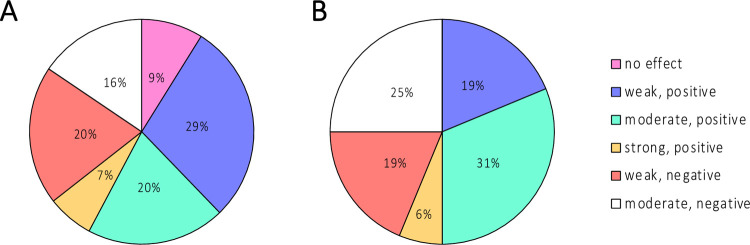
Effect of co-colonizing CoNS on *S*. *aureus* biofilm biomass in co-culture biofilms in (A) cases and (B) controls. The effect was calculated based on log_2_ fold change from *S*. *aureus* mono-species biofilm biomass. Effect on biofilm biomass was arbitrarily classified based on log_2_ fold change as follows: “no effect” (> -0.1 to <0.1); “weak, positive” (0.1 to <0.4); “moderate, positive” (0.4 to <0.9); “strong, positive” (>0.9); “weak, negative” (-0.1 to < -0.4); “moderate, negative” (-0.4 to < -0.9).

**Fig 6:**
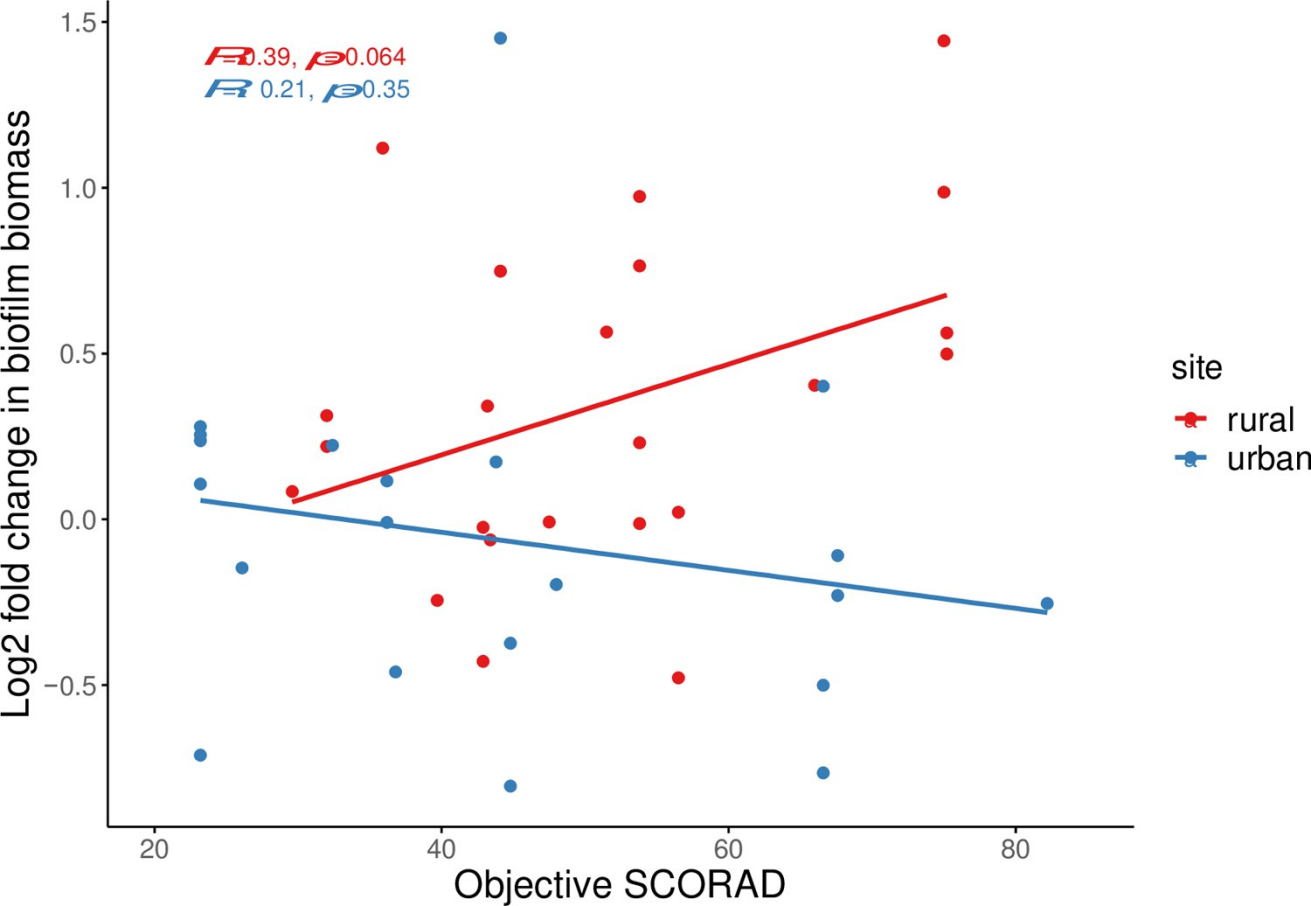
Pearson’s correlation between objective SCORAD and the log fold change of biofilm biomass in co-biofilm cultures of CoNS and *S*. *aureus* from mono-species *S*. *aureus* biofilms in rural and urban cases.

Due to the low numbers of certain species, we opted to report the overall *S*. *aureus* and CoNS biofilm phenotypes without stratifying by species. We observed an overall modest-to-high prevalence of strong biofilm-producing-staphylococci (43%-72%) regardless of disease status, sampling site, or geographic location (Fig 4). Furthermore, *S*. *aureus* and CoNS isolates that lack the ability to form biofilms were only identified in the rural cohort. For 61 *S*. *aureus*-CoNS pairs in co-culture biofilms, we assessed the change in the co-culture biofilm biomass compared to *S*. *aureus* mono-culture biofilm biomass to determine the effect of CoNS on *S*. *aureus* biofilm biomass (Fig 5). We observed no difference in the biofilm biomass of mixed biofilms compared to mono-species *S*. *aureus* biofilms between cases and controls when the geographic location was not considered. Furthermore, there was no relationship between the biofilm biomass fold change in mixed biofilms and disease severity, independent of geographic location (Fig 6).

## Discussion

This study examined staphylococcal colonization and biofilm formation in AD and healthy toddlers from the urban and rural settings in South Africa. Our results showed that AD cases are more commonly colonized by CoNS than controls, although this was mainly limited to nonlesional skin. Also, this study provided evidence that co-colonization with CoNS and *S*. *aureus* on nonlesional skin and anterior nares is associated with more severe disease. However, this was dependent on rural-urban living. Staphylococcal biofilm formation did not differ between cases and controls and might not be significant in staphylococcal pathogenicity in AD.

We observed that nonlesional skin colonization with *S*. *capitis* and *S*. *haemolyticus* was more common in urban and rural cases, respectively, compared to their control counterparts. This observation is similar to previous reports that showed increased abundance of these two CoNS species in cases compared to controls [15]. A recent randomized phase I clinical trial revealed that *S*. *hominis* is a protective CoNS, especially against *S*. *aureus* colonization, and improved AD disease scores [33]. The prevalence of *S*. *hominis* is generally reduced in AD lesions, especially in severe AD, compared to nonlesional skin of both cases and controls [15, 34, 35]. In contrast, some studies noted an increase in its prevalence in AD [36] and no difference in another investigation [37]. In our study, colonization with *S*. *hominis* on nonlesional skin was comparable between cases and controls, independent of geographic location. However, lesional skin was less frequently colonized with *S*. *hominis* than nonlesional skin of cases as previously noted [34], although this was limited to urban participants. These findings support the notion that *S*. *hominis* is primarily protective and is depleted in AD. Moreover, the differences in observation based on location suggest a potential influence of the environment in colonization dynamics. Rural and urban living has been shown to strongly influence the diversity of the skin bacteriome, particularly in early life [38]. This may be due to distinct environmental exposures in rural and urban settings and degrees of interaction with the environment [39]. Specifically, rural living is associated with more diverse skin bacteriomes than urban counterparts [40]. Therefore, we posit that rural living supports the acquisition of a more diverse skin bacteriome [41] and encourages the survival of “protective CoNS” despite AD disease. In contrast, these “protective CoNS” are limited in individuals living in urban areas, and their absence is particularly experienced in AD cases where *S*. *aureus* growth is uninhibited [42].

The prevailing inflammatory and immunological profile in AD has also been shown to influence staphylococcal colonization, with Th2- and Th17-dominance shown to promote *S*. *aureus* growth, especially in severe disease [43]. In contrast, CoNS generally encourages anti-inflammatory skin responses [44], and inflammation in AD inhibits CoNS growth [22]. Moreover, the prevailing immunological profiles in AD have been shown to differ based on rural and urban living, with rural living frequently associated with microinflammation compared to urban living [15, 45, 46]. These observations possibly explain the differences in CoNS colonization and their association with severity between rural and urban toddlers. Collectively, these findings highlight that although some CoNS species may be commensals that are beneficial on healthy skin, they can adapt and increase AD lesions consequently contributing to disease severity. In contrast, other CoNS, such as *S*. *hominis*, are inhibited. The mechanisms by which this adaption, or lack thereof, occur are not clear and therefore warrant further study.

Blicharz *et al*. [47] showed that adult patients with AD co-colonized with *S*. *aureus* and CoNS on the skin and anterior nares had lower IgE levels than those colonized only with *S*. *aureus*. This trend negatively correlates with AD severity [48, 49], suggesting that *S*. *aureus* and CoNS co-colonization relates to less severe disease. However, this might depend on the co-colonizing CoNS species or strains. A positive correlation between disease severity and the simultaneous colonization of CoNS and *S*. *aureus* has been demonstrated [34]. We observed that cases co-colonized with *S*. *aureus* and CoNS on lesional, nonlesional skin, or anterior nares had higher disease severity scores. However, this observation depended on geographic location. It has been suggested that the concurrent increase in *S*. *aureus* and CoNS colonization reflects a compensatory effect of the CoNS in response to *S*. *aureus* proliferation in AD, in particular severe AD [3]. In contrast, CoNS may cooperate with *S*. *aureus* promoting its deleterious effects in AD [13, 50]. Nonetheless, our analysis is limited to correlating the simultaneous staphylococcal growth and AD severity. Therefore, future studies are needed to assess the clinical relevance of this co-colonization and how it contributes to AD flares and disease severity.

Data on the relationship between the colonization of specific CoNS species on AD skin and disease severity is inconsistent [34, 43, 51]. Our study observed that colonization with *S*. *capitis* on nonlesional skin and anterior nares in rural and urban cases was positively associated with higher severity scores than those not colonized. This observation is consistent with Edslev *et al*. [34] who noted a positive association between *S*. *capitis* colonization and AD severity. However, an absence of correlation has also been demonstrated [43]. Our findings suggest that *S*. *capitis* in this cohort directly contributes too AD pathogenesis through mechanisms not been described in the literature. Furthermore, like previous reports, we observed no relationship between *S*. *hominis* and severity scores independent of colonization site and geographic location [43]. Our findings contrast other reports that reported a negative correlation between *S*. *hominis* and disease severity [34]. These conflicting findings could also indicate differences at the host level [52], which may differ across ethnicities, age groups, or geographies, affecting bacterial colonization in skin diseases [53] and their association with disease severity. Overall, our results suggest that the relationship between CoNS and AD severity depends on the colonizing CoNS species.

The disrupted skin barrier in AD lesions exposes the underlying matrix components to which staphylococci bind thereby promoting staphylococcal adhesion to the epidermis [20, 54]. Staphylococcal binding is followed by biofilm formation, which leads to persistent colonization [55]. Patients with AD are frequently colonized by strong biofilm-producing *S*. *aureus* and *S*. *epidermidis* strains, which is associated with more severe disease [26, 39]. We noted a modest-to-high prevalence of strong biofilm-producing staphylococcal isolates, which did not differ between cases and controls, regardless of sampling site and geographic location. CoNS may antagonize or synergize with *S*. *aureus* in mixed biofilm cultures [12, 27]. However, the link between these interactions and AD pathogenesis remains poorly understood and limited to patients with mild AD [26]. Compared to mono-*S*. *aureus* biofilms, we found no overall difference in the fold change of mixed biofilms of co-colonizing *S*. *aureus* and CoNS in cases and controls. Also, this was not associated with disease severity independent of geographic location. Collectively, these findings suggest that the ability to forms biofilms and the outcome of these interactions are innate features of CoNS and *S*. *aureus* and may not relate to disease pathogenesis. Of note, the AD skin environment may alter the ability of staphylococci to form biofilms *in vivo*. For example, alkaline pH, a common feature of AD skin [56], and the cathelicidin LL-37 [57] hamper staphylococcal biofilm formation *in vitro* [58]. Moreover, *S*. *aureus* and *S*. *epidermidis* grow differently in dual-species biofilms at high pH *in vitro* [59], affecting their cooperation in biofilm growth and, consequently, biofilm biomass. In contrast, other host factors, including the pro-inflammatory environment in AD, can augment staphylococcal biofilm formation [22]. The differential effect of various AD disease features on staphylococcal biofilm formation warrants the need for *in vivo* studies. These investigations would include studies using murine [60] or *ex vivo* human skin [61] models of healthy and AD skin to elucidate the dynamics of staphylococcal biofilm formation in AD and how these contribute to disease parameters.

## Strengths and limitations

The strengths of this study include the following. Firstly, a unique rural and urban cohort provided insights into how staphylococcal colonization differs between cases and controls in toddlers across geographies with distinct environmental exposures. Secondly, although *S*. *epidermidis* is the most dominant and studied skin CoNS in AD, we expanded our analyses to other CoNS species on the skin and anterior nares. Thus, the analyses provided a comprehensive characterization of the colonization and biofilm propensity of the staphylococci and how they associate with disease severity in early childhood AD. Nonetheless, our study has some limitations. These include using culture-dependent approaches to describe staphylococcal colonization patterns and biofilm formation. These methods may underrepresent the staphylococcal community due to reliance on bacterial viability and observable colonies formed on solid media. Moreover, we focused our analyses on the presence/absence of staphylococci from the skin and nasal samples. This does not evaluate differences in the abundance (based on colony-forming units) of the staphylococci and its correlation with measures of diseases. The cross-sectional design prevents the assessment of the causality between staphylococcal colonization and biofilm phenotypes with AD severity. Although all cases had used emollients before sampling, we could not account for the timing of emollient administration before sampling. This scenario may have affected the patterns of staphylococcal colonization presently observed [62] and may explain the modest prevalence of CoNS species on lesional skin compared to other studies. Moreover, the presently found differences were by chance due to our present small sample size coupled with the multiple levels of stratification by disease status, geographic location, and sampling site. Therefore, we advocate for future studies which will investigate CoNS colonization in AD in a larger cohort. Despite these limitations, our study provides a unique knowledge on skin and nasal staphylococcal colonization, biofilm propensity, their association with disease severity in early childhood AD, and how these factors differ based on rural-urban living.

## Conclusion

In summary, although CoNS colonization is generally beneficial on healthy skin, we show that CoNS, particularly *S*. *capitis*, are associated with AD severity. This observation could play a role in the pathogenesis and exacerbation of AD through mechanisms not yet fully described in the literature. Furthermore, this may be dependent on their interactions with *S*. *aureus*. Our study highlights that while *S*. *aureus* remains the most studied *Staphylococcus* species in the pathogenesis of AD, CoNS may contribute to AD exacerbations. It also highlights the need for further *in vivo* studies on specific CoNS species or strains and how they contribute to AD exacerbation. Understanding the dynamics of the pathological role of CoNS will allow their targeting by therapeutic strategies aimed at countering complications from CoNS-associated infections in AD.

## Supporting information

S1 Table Unconditional logistic regression analysis of child, parental, domestic and environmental characteristics associated with CoNS colonization in Umtata and Cape Town participants. (DOCX)
