# Supplementary material for: Skin and nasal colonization of coagulase-negative staphylococci are associated with atopic dermatitis among South African toddlers
Source: PLoS One. 2022 Mar 17;17(3):e0265326. doi: 10.1371/journal.pone.0265326 (PMC8929619; doi:10.1371/journal.pone.0265326)
Supplement: S1 Table — (DOCX) [file pone.0265326.s001.docx]

Table S1. Unconditional logistic regression analysis of child, parental, domestic and environmental characteristics associated with CoNS colonisation in Umtata and Cape Town participants

| Explanatory variable | Umtata (rural) | | | | Cape Town (urban) | | | |
| --- | --- | --- | --- | --- | --- | --- | --- | --- |
|  | OR [95% CI] | *p*-value | aOR [95% CI] ^a^ | *p*-value | OR [95% CI] | *p*-value | aOR [95% CI] ^a^ | *p*-value |
| AD: case | 4.14 [1.83-9.35] | **0.001** | 2.55 [0.92-7.06] | 0.071 | 3.6 [1.36-9.51] | **0.010** | 4.67 [1.58-13.77] | **0.005** |
| Sex: male | 0.70 [0.31-1.57] | 0.385 | Removed |  | 0.71 [0.28-1.81] | 0.479 | Removed |  |
| Child characteristics |  |  |  |  |  |  |  |  |
| Allergic rhinitis | 1.89 [0.34-10.33] | 0.463 | Removed |  | Omitted |  |  |  |
| Asthma | Omitted |  |  |  | Omitted |  |  |  |
| Food allergy | 1.00 [0.27-3.63] | 0.995 | Removed |  | 5.32 [0.64-44.40] | 0.123 | Removed |  |
| Breastfeeding | 0.67 [0.29-1.51] | 0.332 | Removed |  | 1.64 [0.40-6.62] | 0.490 | Removed |  |
| Mode of delivery: vaginal | 1.71 [0.70-4.18] | 0.236 | Removed |  | 0.55 [0.21-1.46] | 0.231 | Removed |  |
| Incomplete immunisation status ^d^ | 1.74 [1.17-17.28] | 0.637 | Removed |  | 6.05 [0.73-50.00] | 0.095 | Removed |  |
| Antibiotic exposure | 1.21 [0.45-3.26] | 0.703 | Removed |  | 3.99 [1.44-11.09] | **0.008** | 5.25 [1.68-16.38] | **0.004** |
| Large family | 0.79 [0.37-1.71] | 0.557 | Removed |  | 0.92 [0.30-2.82] | 0.879 | Removed |  |
| Farm animal exposure | 0.17 [0.04-0.76] | **0.021** | 0.35 [0.07-1.80] | 0.207 | Omitted |  |  |  |
| Fossil fuel exposure |  |  |  |  |  |  |  |  |
| Electricity + gas | 1.84 [0.84-4.05] | 0.130 | Removed |  | Omitted |  |  |  |
| Kerosene + paraffin | 3.01 [1.36-6.66] | **0.007** | 2.59 [0.84-8.01] | 0.099 | 0.92 [0.34-2.45] | 0.870 | Removed |  |
| Indoor fire | 1.74 [0.17-17.28] | 0.637 |  |  | Omitted |  |  |  |
| Outdoor fire | 0.40 [0.18-0.87] | **0.022** | 0.76 [0.29-1.95] | 0.565 | Omitted |  |  |  |
| Wood + coal | 0.41 [0.18-0.96] | **0.040** | 1.79 [0.49-6.58] | 0.380 | Omitted |  |  |  |
| Maternal factors |  |  |  |  |  |  |  |  |
| Allergic rhinitis | 1.74 [0.17-17.28] | 0.637 | Removed |  | Omitted |  |  |  |
| Asthma | Omitted |  |  |  | 2.77 [0.31-24.96] | 0.365 | Removed |  |
| Atopic dermatitis | Omitted |  |  |  | Omitted |  |  |  |
| Food allergy | Omitted |  |  |  | Omitted |  |  |  |
| Smoking | Omitted |  |  |  | 1.77 [0.17-17.94] | 0.628 | Removed |  |
| Pregnant smoker | Omitted |  |  |  | 1.12 [0.10-12.96] | 0.930 | Removed |  |
| Animal exposure | 0.39 [0.10-1.47] | 0.163 | Removed |  | 0.51 [0.07-3.88] | 0.517 |  |  |
| Paternal factors |  |  |  |  |  |  |  |  |
| Allergic rhinitis | 1.14 [0.10-13.00] | 0.914 | Removed |  | 4.04 [0.47-34.73] | 0.203 | Removed |  |
| Asthma | 1.14 [0.10-13.00] | 0.914 | Removed |  | Omitted |  |  |  |
| Atopic dermatitis | Omitted |  |  |  | Omitted |  |  |  |
| Food allergy | Omitted |  |  |  | Omitted |  |  |  |
| Smoking | 0.59 [0.20-1.77] | 0.345 | Removed |  | 1.43 [0.47-4.36] | 0.529 | Removed |  |

^a^ Variables without statistical significance in the univariate model were excluded from the multivariate model. ^b^ Large family was arbitrarily defined by more than six members within a household. ^c^ Variables omitted due to dependency in the regression model. ^d^ Immunisation to routine childhood vaccines. Abbreviations: AD, atopic dermatitis; OR, odds ratio; CI, confidence interval.
